# Markerless head motion tracking and event-by-event correction in brain PET

**DOI:** 10.1088/1361-6560/ad0e37

**Published:** 2023-12-12

**Authors:** Tianyi Zeng, Yihuan Lu, Weize Jiang, Jiaxu Zheng, Jiazhen Zhang, Paul Gravel, Qianqian Wan, Kathryn Fontaine, Tim Mulnix, Yulin Jiang, Zhaohui Yang, Enette Mae Revilla, Mika Naganawa, Takuya Toyonaga, Shannan Henry, Xinyue Zhang, Tuoyu Cao, Lingzhi Hu, Richard E Carson

**Affiliations:** 1 Department of Radiology and Biomedical Imaging, Yale University, New Haven, CT, United States of America; 2 United Imaging Healthcare, Houston, TX, United States of America

**Keywords:** markerless motion tracking system, PET, head motion, event-by-event correction, registration quality metric

## Abstract

*Objective.* Head motion correction (MC) is an essential process in brain positron emission tomography (PET) imaging. We have used the Polaris Vicra, an optical hardware-based motion tracking (HMT) device, for PET head MC. However, this requires attachment of a marker to the subject’s head. Markerless HMT (MLMT) methods are more convenient for clinical translation than HMT with external markers. In this study, we validated the United Imaging Healthcare motion tracking (UMT) MLMT system using phantom and human point source studies, and tested its effectiveness on eight ^18^F-FPEB and four ^11^C-LSN3172176 human studies, with frame-based region of interest (ROI) analysis. We also proposed an evaluation metric, registration quality (*RQ*), and compared it to a data-driven evaluation method, motion-corrected centroid-of-distribution (MCCOD). *Approach.* UMT utilized a stereovision camera with infrared structured light to capture the subject’s real-time 3D facial surface. Each point cloud, acquired at up to 30 Hz, was registered to the reference cloud using a rigid-body iterative closest point registration algorithm. *Main results.* In the phantom point source study, UMT exhibited superior reconstruction results than the Vicra with higher spatial resolution (0.35 ± 0.27 mm) and smaller residual displacements (0.12 ± 0.10 mm). In the human point source study, UMT achieved comparable performance as Vicra on spatial resolution with lower noise. Moreover, UMT achieved comparable ROI values as Vicra for all the human studies, with negligible mean standard uptake value differences, while no MC results showed significant negative bias. The *RQ* evaluation metric demonstrated the effectiveness of UMT and yielded comparable results to MCCOD. *Significance.* We performed an initial validation of a commercial MLMT system against the Vicra. Generally, UMT achieved comparable motion-tracking results in all studies and the effectiveness of UMT-based MC was demonstrated.

## Introduction

Motion tracking methods for brain positron emission tomography (PET) can be categorized into data-driven and hardware-based motion tracking (HMT) categories. Data-driven methods use PET raw data or reconstructions and do not require external devices. The post-reconstruction registration method employs pre-defined dynamic frames, which are registered to a reference frame to obtain motion transformations (Mukherjee *et al* 2016, Picard and Thompson 1997). Sun *et al* utilized tracer-specific kinetic modeling to deal with inter-frame movement patterns for dynamic PET (Sun *et al* 2022b). However, intra-frame motion, i.e. motion within one dynamic frame, cannot be corrected. Data-driven methods using PET raw count data, such as centroid of distribution (COD) and moments of inertia, can achieve great reductions in motion-induced blurring, but generally do not have high temporal resolution and can be inaccurate during large changes in tracer activity (Schleyer *et al* 2015, Rezaei *et al* 2021, Revilla *et al* 2022). Recently, deep learning for head motion correction (DL-HMC) has demonstrated its feasibility in predicting rigid motion for brain PET (Zeng *et al* 2022), but further advancements are necessary to enhance its robustness.

**Figure 1.**
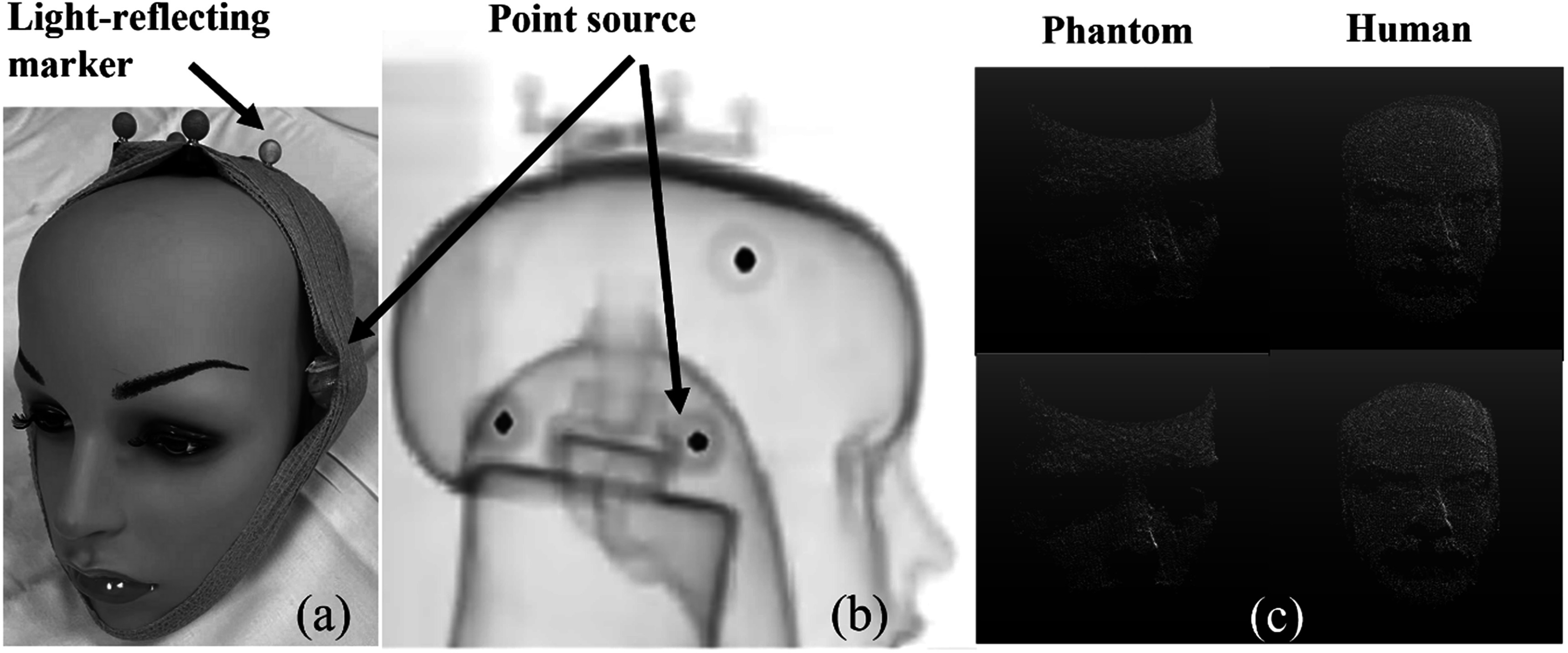
(a) Mannequin phantom with light-reflecting marker and radioactive point sources, (b) Overlay between the maximum-intensity-projection of the phantom attenuation map and the PET reconstruction of the point sources, (c) example of a phantom point cloud (left) before registration (top) and after registration (bottom) and example of a patient point cloud (right) before registration (top) and after registration (bottom).

Compared to data-driven motion tracking, HMT typically has higher temporal and spatial resolution. For example, in Onishi *et al* (2022), a brain PET scanner has an inter-crystal gap for a retro-reflective marker-based HMT. At our center, the Polaris Vicra (Northern Digital Inc.), an optical HMT device mounted outside the scanner, has been used in over 5000 PET studies (Jin *et al* 2013b). However, Vicra is not routinely used clinically due to the requirement to attach a light-reflecting marker (figure 1(a)) to the patient. Also, slippage of the attached markers can occur.

Compared to marker-based HMT, markerless motion tracking (MLMT) methods are more convenient. In MLMT, camera images are captured to detect head movements in real time. The core principle of MLMT involves employing computer vision techniques for robust feature matching, enabling the computation of changes in head position accurately. In the past, MLMT has been successfully applied in MRI motion tracking (Kyme *et al* 2020, Chen *et al* 2023). Over time, several markerless motion tracking methods for PET have been proposed. In animal studies, Kyme *et al* (2014) and Miranda *et al* (2017) applied MLMT to track head motion in awake rodents and successfully demonstrated its effectiveness in correcting motion in rat PET. In human studies, Olesen *et al* (2013) applied markerless tracking at 5 Hz for brain PET using two cameras, where the reference images were created by aligning and merging point clouds from two cameras, and the tracking result was calculated by the iterative closest point (ICP) algorithm; in a subsequent paper (Slipsager *et al* 2019), an improved 30 Hz MLMT with one camera was applied in both PET and magnetic resonance imaging (MRI), also using ICP registration to obtain motion information, and the motion correction effectiveness was evaluated in a large cohort study by time-activity curve analysis. However, these studies did not include a comparison study with other motion tracking methods. Iwao *et al* (2022) applied a time-of-flight type range sensor in a helmet PET system and achieved event-by-event (EBE) head motion correction (MC), although quantitative accuracy in the final images was not yet evaluated. In addition, note that the proposed Kinect system was designed for helmet geometry and would require adaptation for cylindrical PET geometries. Overall, current MLMT methods lack validation, and evaluation metrics are limited. Therefore, even with encouraging initial results, there is not yet a robust commercial system for brain PET motion correction.

Careful evaluation of the MC method is necessary. Ideal MC evaluation methods, such as direct measurements of motion and comparison of motion displacement to ground truth, are not clinically feasible. Comparison of frame-based standardized uptake value (SUV) is a common MC evaluation method. Several other evaluation methods such as contrast-to-noise ratio (CNR), cross correlation, mutual information and standardized uptake value ratio (SUVR) were also proposed for different motion correction applications (Klen *et al* 2016, Chen *et al* 2018, Keller *et al* 2012, Reilhac *et al* 2018). We recently proposed an objective quality control metric for rigid head motion information, called motion corrected centroid-of-distribution (MCCOD) (Sun *et al* 2022a). MCCOD has the advantage over other methods of providing real-time assessment of motion data accuracy. Such real-time data provides the opportunity to correct or remove periods of large motion where registration accuracy may be poor. In markerless HMT, detection accuracy relies on device robustness, such as registration between moving frames and segmented face models, and accuracy is subject to non-rigid facial expression changes (Iwao *et al* 2022). Therefore, a quantitative evaluation metric for MLMT is important and should be objective and real-time.

In this study, we propose a prototype markerless HMT camera developed by United Imaging Healthcare (UIH), to perform real-time head motion tracking in brain PET. We validate the UIH motion tracking (UMT) system against the Vicra (1) using phantom and human studies with attached radioactive point sources, and (2) with human ^18^F-FPEB and ^11^C-LSN3172176 studies. We also test a camera-based evaluation metric (registration quality, *RQ*) and apply MCCOD for comparison.

## Materials and methods

### Human studies

Subjects were enrolled in institutional review board–approved studies that were also approved by the Yale Radiation Safety Committee. Subjects were also enrolled in a separate institutional review board–approved protocol for evaluation of the camera system. All subjects gave written informed consent.

### Data acquisitions

All studies were performed on a Siemens Biograph mCT (Jakoby *et al* 2011) using both the Vicra and UMT systems. The UMT was mounted on the gantry of the scanner, and time synchronization was applied between UMT and mCT (see Supplement). In a 15 min phantom study, three Na-22 point sources (80 kBq each) and the Vicra tool were attached to a mannequin head (figures 1(a), (b)). Thirteen manual translation and rotation step motions were performed every minute, and the phantom was held static between movements. CT was used for attenuation correction (AC). The phantom was stationary during the CT acquisition and the first minute of the PET scan.

A 15 min human study mimicking the phantom study was performed with the same point sources and without tracer injection. A volunteer was instructed to perform step head motions of translation and/or rotation every minute. The volunteer was instructed to remain static for the first minute and between step motions. No CT was acquired.

As part of ongoing studies, twelve 120 min PET dynamic human studies were performed using ^18^F-FPEB (Wong *et al* 2013, Mecca *et al* 2021) (153 ± 24 MBq injection, metabotropic glutamate 5 receptors, *N* = 8) and ^11^C-LSN3172176 (500 ± 209 MBq, muscarinic M1 receptor, *N* = 4) (Naganawa *et al* 2021). CT acquired prior to the PET was used for AC. Each participant underwent separate T1-weighted MRI.

### Motion tracking methods

UMT utilized a stereovision camera with infrared structured light to capture the subject’s real-time 3D facial surface. The system consisted of an illumination class I laser (940 nm) and two infrared cameras which collected reflected optical signal. These data were transferred to a dedicated processor to calculate point clouds (a list of spatial coordinates) at up to 30 Hz. There were ∼20 000 points per cloud, with spatial accuracy <0.2 mm as validated by a high-resolution motion stage. The point clouds were down-sampled by averaging point location within a grid of 3 mm voxels (Steinbrucker *et al* 2014).

To perform motion estimation, each point cloud (figure 1(c)) was registered to a reference point cloud collected at the beginning of each scan, using a rigid-body ICP registration algorithm (Besl and Mckay 1992). The average calculation time for a 120 min human protocol was approximately 30 min. This algorithm found a 6 degree-of-freedom solution to minimize the sum of the squared distance between points in each point cloud pair (Chen and Medioni 1992). A bounding box was manually selected and applied on the first frame, and the reference cloud was segmented within the bounding box to include the upper face/cheek area and exclude all non-face areas. During motion tracking, the ICP algorithm was only applied within the bounding box. If the rotation or translation output from the ICP registration exceeded empirically set thresholds (3 milliradian rotation or 0.5 mm translation in any direction), the current moving frame was considered to contain significant motion. Subsequently, the bounding box was repositioned based on the transformation matrix and applied to subsequent frames. The final transformation matrix was converted to the PET coordinate system via a pre-calculated calibration matrix (see Supplement). No filtering was applied to the motion data.

The UMT framework was compared with the Vicra, which was used as the reference. The Polaris Vicra tracking system (Northern Digital Inc., Waterloo, Canada) used infrared illuminators and stereo cameras to sense 3D positions of reflective spheres, which were mounted to the subject’s head with a ‘tool’. Data were collected at 30 Hz.

To measure head motion between CT and PET, a second Vicra reflection tool (‘bed tool’) was attached to the patient bed and was tracked along with the tool on the patient’s head (‘head tool’). We assumed that the patient’s head was motionless relative to the bed during the CT acquisition, so the spatial relationship of the head tool relative to the bed tool was constant, i.e. a fixed transformation matrix. With the bed tool, Vicra can track the motion from CT pose to PET pose to eliminate attenuation mismatch. For this evaluation, the transformation matrix between CT and PET poses was applied in the proposed UMT framework, since UMT did not measure the motion between PET and CT (see Supplement). In the future, we will develop CT automatic segmentation and registration algorithms for UMT.

### Event-by-event motion compensated reconstruction and image analysis

Image reconstruction was performed using the MOLAR (Motion-compensation OSEM list-mode algorithm for resolution-recovery reconstruction) platform (Jin *et al* 2013a). Motion information at 30 Hz provided by Vicra or UMT was converted to a text file with one line for each measurement, including time tag and the 12 values in the rigid transformation matrix. Then, EBE MC reconstructions were performed by reassigning the endpoints of each line-of-response (LOR) according to the motion information. Both methods used the same reconstruction pipeline and frame timing. OSEM reconstruction (3 iterations × 21 subsets) with spatially invariant point-spread-function (PSF) of 4 mm full-width-half-maximum (FWHM) was used (Jin *et al* 2013a). Time-of-flight information was included in the reconstruction and no post-smoothing filter was applied. An isotropic voxel size of 0.5 mm was used for the point source reconstructions and 2 mm was used for the ^18^F-FPEB and ^11^C-LSN3172176 studies. Dynamic PET data were reconstructed into 33 frames: 6 × 30 s, 3 ×1 min, 2 ×2 min, 22 ×5 min. For the phantom and human point source studies, the six parameters of rigid motion measured by the Vicra and UMT systems were compared. A full 15 min frame and fifteen 1 min frames were reconstructed. To assess within-image blurring, the regions around each point source were fitted to a three-dimensional (X: lateral, Y: anterior-posterior, and Z: axial), Gaussian model with 8 parameters (peak height, background, X, Y, and Z center locations, and X, Y, and Z FWHMs). Measurements made in the reconstruction of the 1st minute, i.e. no motion, were used as the reference. Center shift of the point sources was measured by the Euclidian distance (mm) of the estimated centers between target and reference reconstructions. For the ^18^F-FPEB and ^11^C-LSN3172176 human studies, region of interest (ROI) analysis was applied (see Supplement). For each ROI, paired sample t-tests (one-tailed) were applied to the absolute value of the percent differences with respect to the Vicra data, to test statistically if the UMT error was smaller than that of the no motion correction data.

### Motion correction evaluation methods

Two motion correction metrics were evaluated. The first metric was based solely on motion data. Due to head motion and facial expression changes, e.g. mouth breathing, ICP registration may be inaccurate. To quantify the UMT MC quality, we proposed a metric called registration quality (*RQ*), which quantified registration accuracy as the fraction of points in the point cloud that were accurately registered:

\begin{eqnarray*}\mathrm{RQ}=\frac{N(m^{\prime} )}{N(m)}\end{eqnarray*}

\begin{eqnarray*}{m}^{{\prime} }\in \left\{{m}_{i}\right|\varphi \left({m}_{i},{r}_{i}\right)\leqslant {d}^{2}\},\end{eqnarray*}

where *N* is the number of points (∼5000 points per cloud), $m$ represents the registered moving frame, $r$ represents the reference frame, and $i$ indexes the points in the point cloud. $\varphi $ is a squared Euclidean distance operator quantifying the closeness of ${m}_{i}$ and ${r}_{i}.$ The distance threshold, *d*, was empirically set at 2.4 mm (80% of the UMT voxel size). To quantify overall registration quality, the time duration with *RQ* below an empirical threshold (0.97) was tabulated for each study.

The second metric was based on PET raw data. We have previously used COD as a data-driven method to detect motion (Lu *et al* 2019, Ren *et al* 2017). The central coordinates of the LOR of all events were averaged over 1 s intervals to generate COD traces in X, Y, and Z. A follow-on to COD was motion-corrected COD (MCCOD), which was an objective quality control approach to assess rigid motion information (Sun *et al* 2022a); see Supplement for details. MCCOD displacements indicated motion estimation errors.

In the human study, we generated MCCOD traces based on both Vicra and UMT data to qualitatively evaluate UMT motion tracking performance. In addition, we assessed the effectiveness of the *RQ* metric by comparing ‘spikes’ in this trace to large visible MCCOD displacements.

## Results

### Phantom and human study with external point sources

**Figure 2.**
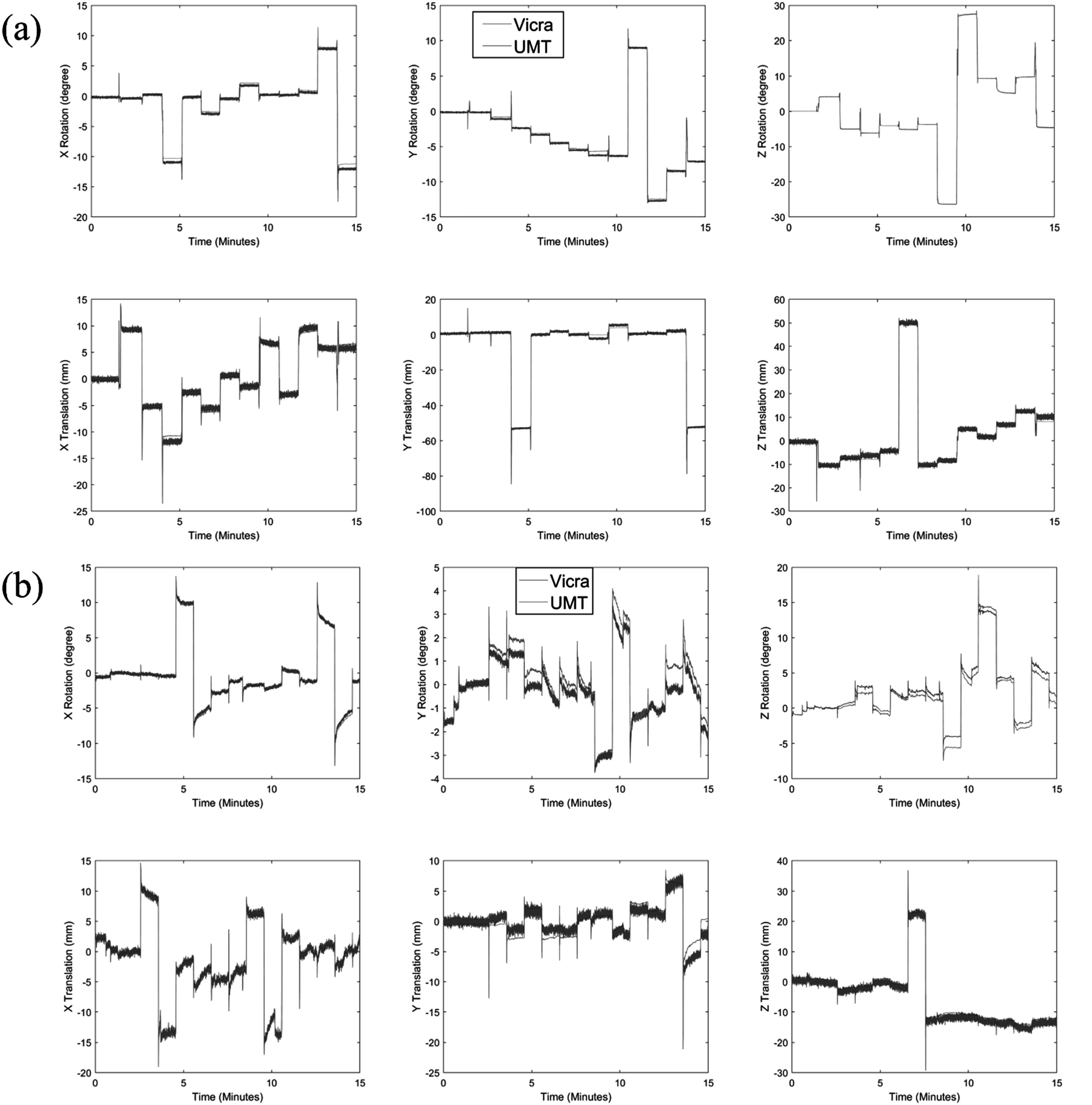
Motion tracking results of point source studies for (a) the mannequin phantom study and (b) the human volunteer study. UMT data are shown in blue and Vicra data are shown in orange. Note the differences in scale. UMT: United Imaging Healthcare Motion tracking system.

For the phantom point source study, the mean and standard deviation (SD) of rotation and translation differences tracked by UMT and Vicra were 0.39 ± 0.71° and −0.60 ± 2.18 mm, respectively. Qualitative results show that UMT and Vicra motion tracking are in an overall very good agreement (figure 2(a)). Small discrepancies in rotation (0.65 ± 0.10°) and translation (1.08 ± 0.28 mm) in *X* at 4–5 min as well as rotation (0.59 ± 0.06°) in *Y* at 8–9 min were observed. Short period disagreements (‘spikes’) between Vicra and UMT occurred at the times of step motions; these may be caused either by the Vicra tool wobbling or by poor performance of the UMT ICP registration. The SD of rotation and translation during the static time periods between step motions for UMT were 0.04° and 0.05 mm, and for Vicra were 0.07° and 0.39 mm, i.e. there were less high-frequency fluctuations between step motions for the UMT than the Vicra; this difference in variability is visible in figure 2(a).

**Figure 3.**
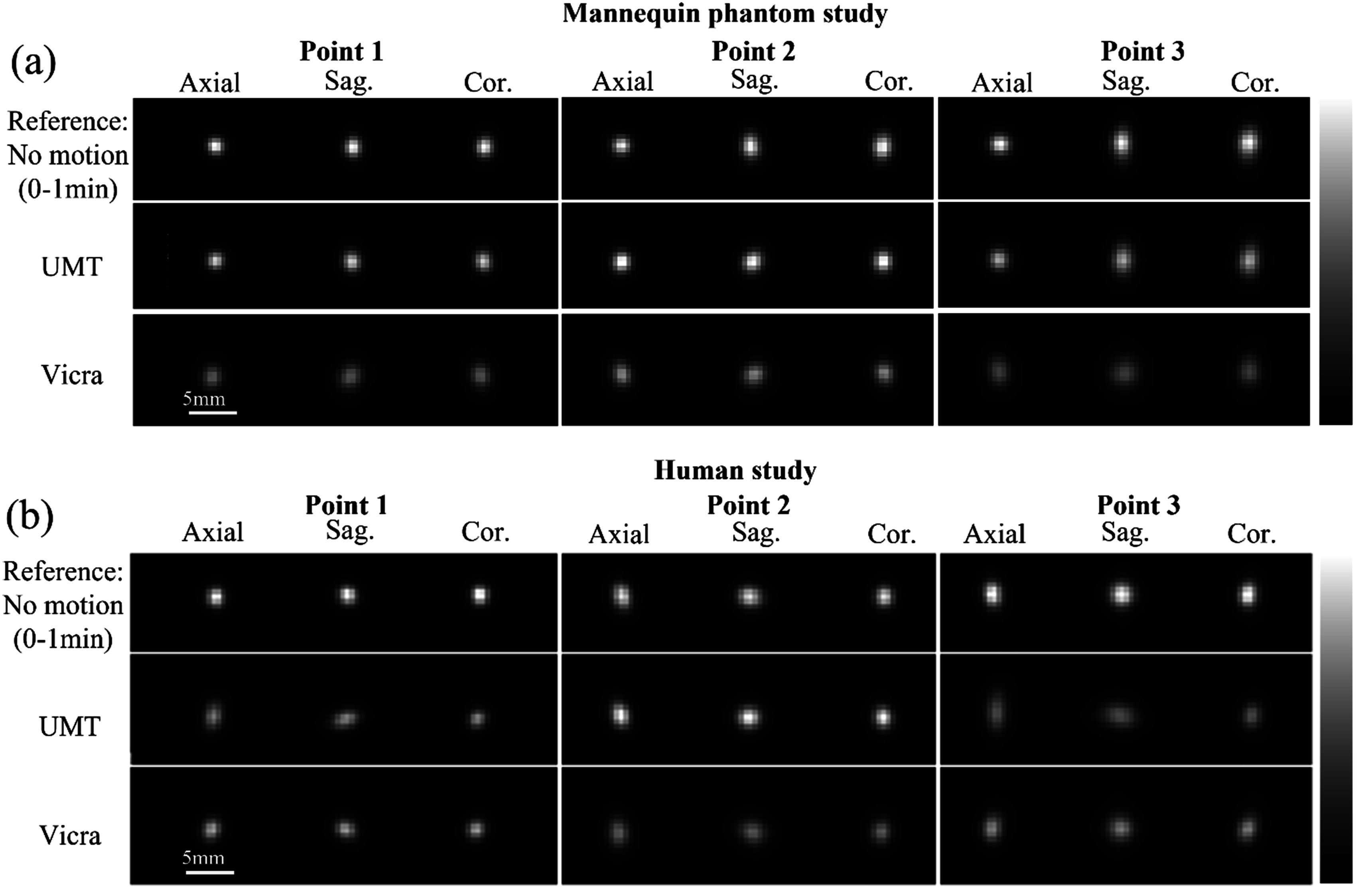
Reconstructed images of the point sources for (a) the mannequin phantom study and (b) the human volunteer study. Slices shown intersect the highest voxel value for each source for each method. The top row shows the reconstruction of the first minute of acquisition (no motion). The next two rows show the full 15 min reconstructions with motion correction by UIH Motion Tracking (UMT) and Vicra. For each point source, the color bar was scaled to the maximum intensity from the reference image, which differs for each source. All the images for each source were displayed to their respective maxima for visual comparison. UMT: United Imaging Healthcare Motion tracking system.

Figure 3(a) shows reconstructed axial, sagittal, and coronal images of the point sources in the phantom study; these slices intersect the highest pixel value for each source for each method. For the 0–1 min reference phase (figure 3(a), top row, no motion), isotropic resolution was observed in the *X*–*Y* (axial) planes for all three points while poorer resolution is observed in the *Z* direction, perhaps caused by the spatially-variant resolution of the mCT (Jakoby *et al* 2011). For phases with motion (figure 3(a), rows 2 and 3, 15 min reconstructions), UMT yielded similar peak intensity as the reference image, while Vicra yielded lower peak values consistent with less-accurate motion compensation. For comparison, no motion corrected (NMC) images are shown as maximum intensity projections in comparison to those for UMT and Vicra (see supplemental figure 2).

Table 1(a) shows the analysis of the full (15 min) and frame-based (individual 1 min periods) reconstructions of the point sources for phantom study. Numerically, for all the points and in all directions, UMT showed smaller FWHM values than Vicra (table 1(a)) in both the full analysis (0.35 ± 0.27 mm) and the frame-based analysis (0.09 ± 0.08 mm). UMT also yielded smaller center shifts than the Vicra (differences of 0.12 ± 0.10 mm in full analysis and 0.57 ± 0.02 mm in frame-based analysis). Due to the nature of the mannequin, i.e. no facial expressions, this study represented the best possible UMT performance.

**Table 1.** Full (15 min) and frame-based point source study evaluation. (a) Mannequin phantom point source study result. (b) Human volunteer point source study result.

Mannequin phantom	Point 1				Point 2				Point 3			
	FWHM-*X* (mm)	*Y*	*Z*	Center shift (mm)	*X*	*Y*	*Z*	Center shift	*X*	*Y*	*Z*	Center shift
**(a)**												
No Motion (ref.)	1.87	1.94	2.43	0.00	1.87	1.88	2.17	0.00	1.96	1.90	2.91	0.00
UMT full	**1.82**	**1.98**	**2.11**	**0.03**	**1.83**	**1.86**	**2.18**	**0.01**	**2.00**	**2.05**	**2.97**	**0.07**
Vicra full	1.98	2.46	2.34	0.24	1.98	2.34	2.52	0.14	2.24	3.02	3.06	0.09
UMT frame Mean (SD)	**1.85**	**1.91**	**2.32**	**0.33**	**1.84**	**1.83**	**2.13**	**0.47**	**2.01**	**1.96**	**2.73**	**0.54**
	**(0.07)**	**(0.11)**	**(0.24)**	**(0.25)**	**(0.04)**	**(0.07)**	**(0.16)**	**(0.28)**	**(0.17)**	**(0.15)**	**(0.32)**	**(0.30)**
Vicra frame Mean												
(SD)	1.88	1.96	2.41	0.92	1.88	1.89	2.41	1.03	2.08	2.05	2.87	1.10
	(0.08)	(0.10)	(0.23)	(0.76)	(0.04)	(0.08)	(0.21)	(0.72)	(0.17)	(0.15)	(0.30)	(0.79)

**(b)**												
Human volunteer	Point 1				Point 2				Point 3			
	FWHM-*X* (mm)	*Y*	*Z*	Center shift (mm)	*X*	*Y*	*Z*	Center shift	*X*	*Y*	*Z*	Center shift

No Motion (ref.)	1.61	1.79	1.72	0.00	1.77	2.38	1.94	0.00	1.75	2.30	2.25	0.00
UMT full	**1.75**	2.62	**1.72**	0.82	**1.77**	**2.28**	**2.03**	0.75	**2.03**	3.66	**2.44**	1.23
Vicra full	1.78	**2.05**	1.84	**0.27**	2.02	2.81	2.37	**0.22**	2.03	**2.65**	2.60	**0.46**
UMT frame Mean	**1.65**	**1.87**	**1.60**	1.32	**1.73**	**2.22**	**2.00**	**0.82**	**1.72**	**2.36**	**2.06**	1.97
(SD)	**(0.04)**	**(0.07)**	**(0.11)**	(1.0)	**(0.06)**	**(0.11)**	**(0.12)**	**(0.50)**	**(0.07)**	**(0.11)**	**(0.11)**	(1.52)
Vicra frame Mean	1.72	2.01	1.83	**0.65**	1.82	2.34	2.22	1.04	1.80	2.42	2.36	**1.31**
(SD)	(0.07)	(0.12)	(0.14)	**(0.34)**	(0.07)	(0.14)	(0.16)	(0.52)	(0.05)	(0.10)	(0.14)	**(1.0)**

Mean and SD of FWHM were calculated across all images. The center shift is the distance between the maximum intensity voxel of the reconstructed images and the reference image. No Motion (ref.) is the result of the 0–1 min static frame. UMT full and Vicra full indicate the full 15 min reconstruction evaluation result. UMT frame and Vicra frame indicate the frame-based evaluation result. Bold font indicates whether UMT or Vicra showed superior performance. FWHM: Full Width at Half Maximum. UMT: United Imaging Healthcare Motion Tracking system.

For the human study with point sources, figure 2(b) shows the translation and rotation tracked by UMT and Vicra. For *X* axis rotation and translation, good agreement between UMT and Vicra was found, with mean ± SD differences of −0.18 ± 0.61° and 0.05 ± 0.93 mm, respectively. For the other motion parameters (*Y* axis rotation and translation, *Z* axis rotation and translation), larger differences were found: 0.34 ± 0.36°, −0.07 ± 1.31 mm, −0.46 ± 0.72° and 1.09 ± 1.33 mm, respectively. Like the mannequin study, UMT yielded lower noise in translation tracking than the Vicra. Figure 3(b) illustrates the full 15 min reconstruction of the point sources in the human study. Visually, UMT outperformed Vicra for Point 2, exhibiting a higher peak intensity. For the other sources, both UMT and Vicra displayed lower peak intensities than the reference, accompanied by elongated tails in the axial and coronal views.

Table 1(b) shows analysis results of the human point source study. In the full analysis, at Point 2 (∼5 cm above the left ear of the volunteer), UMT provided better spatial resolution than the Vicra and yielded similar peak intensity as the reference image. For Point 1 (close to top of head) and Point 3 (∼5 cm above the right ear), UMT yielded comparable spatial resolution as the Vicra in *X* and *Z* directions, but resolution degradation in *Y* was observed for Points 1 and 3 for UMT. In addition, UMT yielded substantially larger center shift (by 0.62 ± 0.13 mm) in the full analysis. In the frame-based analysis, UMT had smaller FWHM values than Vicra for all points (0.15 ± 0.08 mm), similar to the mannequin study. As for center shift, Vicra outperformed UMT for 2 points; the average difference was 0.26 ± 0.45 mm for all points. Compared with the mannequin study, UMT had poorer performance of center shift in the human study, perhaps due to its sensitivity to facial expressions such as mouth/nose movement, hands touching face, etc.

### Human PET studies

**Figure 4.**
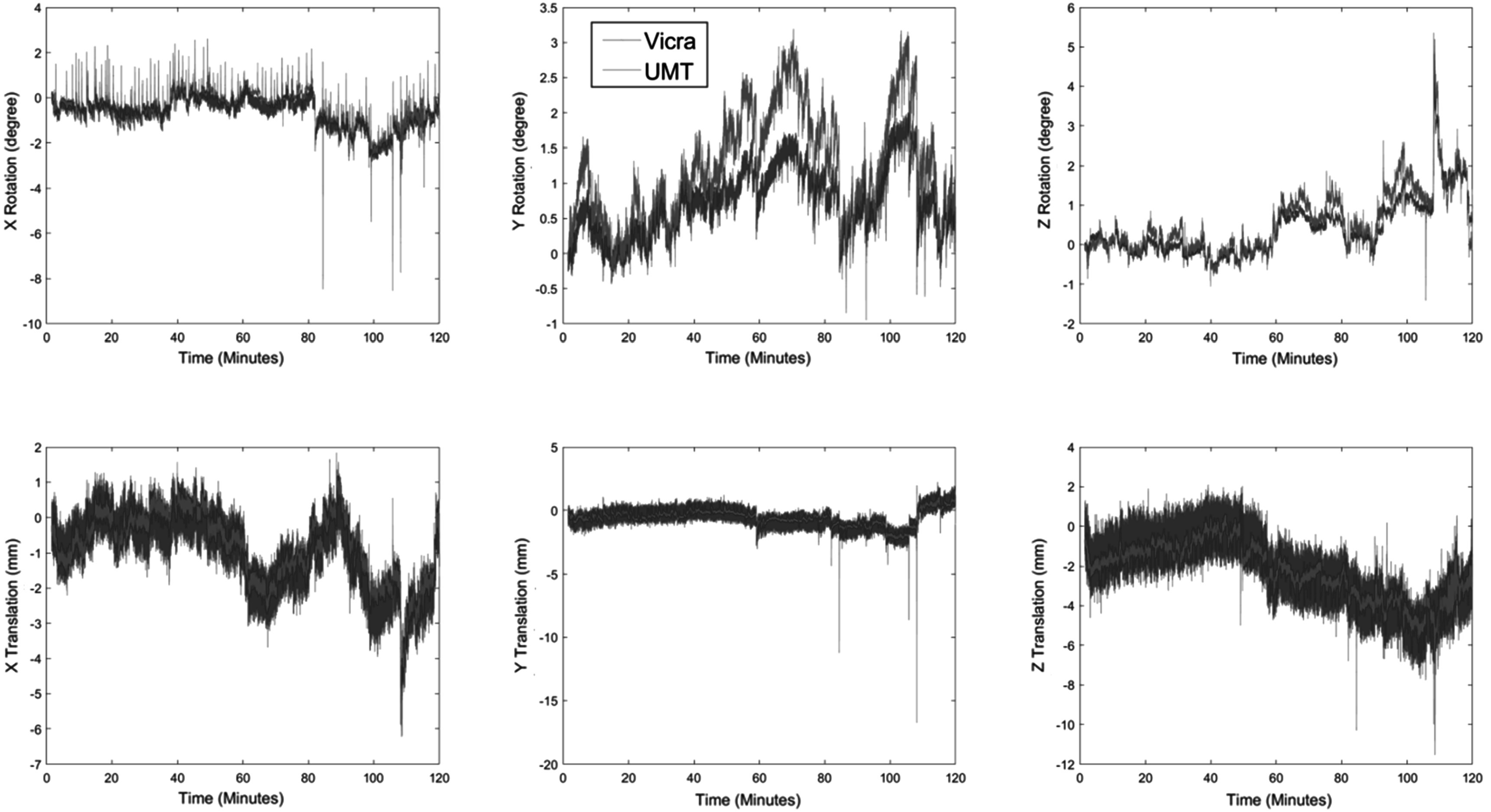
Motion tracking results from one typical ^11^C-LSN3172176 subject. UMT data are shown in orange and Vicra data are shown in blue. Note the differences in scale. UMT: United Imaging Healthcare Motion tracking system.

For the human studies, we compared the real-time motion tracking information from UMT and Vicra in one typical ^11^C-LSN3172176 subject with moderate motion (figure 4). Over a 2 h scan, the mean and SD of rotation and translation differences tracked by UMT and Vicra were 0.84 ± 0.62° and 0.15 ± 0.72 mm, respectively. The translation values in all directions agreed, but there were some minor drifts in *Y* rotations, with mean and SD differences between UMT and Vicra of 0.51 ± 0.35°. These discrepancies may be caused by factors such as nonrigid motion (which can affect UMT) and tool slippage and wobbling (which affects the Vicra). As seen in figure 4, similar to the point source studies, UMT yields lower noise than the Vicra; this is most clearly visualized in the translation results. Quantitatively, we calculated the translation motion SD within 1 min intervals and averaged this value over the 120 min scan across all 12 subjects. The result showed that Vicra average SD was 0.49 mm while UMT was 0.27 mm.

**Figure 5.**
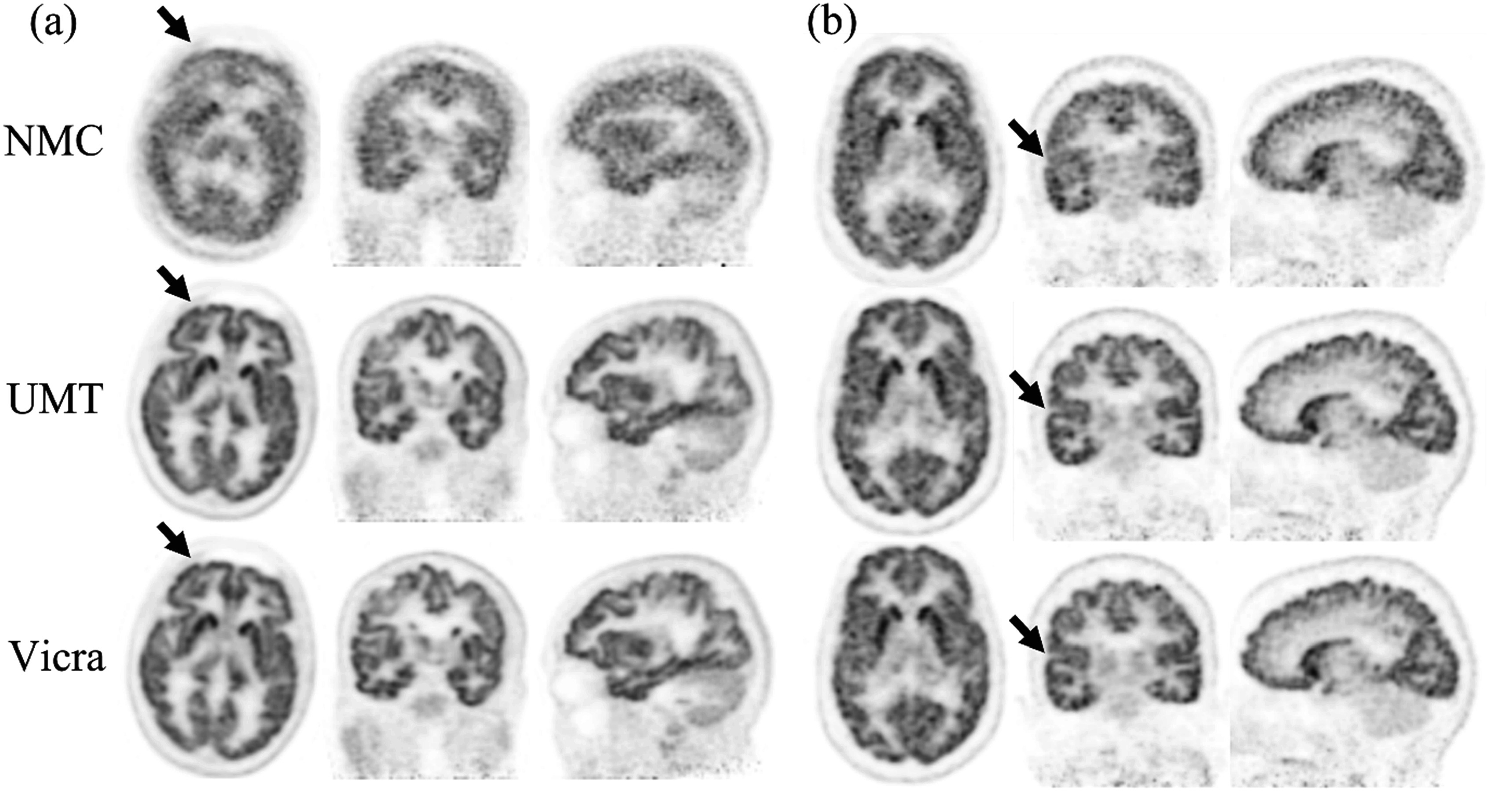
Typical reconstruction examples (0–120 min post injection) of (a) ^18^F-FPEB and (b) ^11^C-LSN3172176. NMC indicates reconstruction without motion correction, UMT indicates reconstruction with UMT-based motion correction, Vicra indicates reconstruction with Vicra-based motion correction. The arrows denote areas of clear difference between UMT and Vicra versus NMC. NMC: No Motion Correction. UMT: United Imaging Healthcare Motion tracking system.

Figure 5 shows reconstructed images for the two tracers with no motion correction (NMC) or motion information from UMT or Vicra. In figure 5(a), compared with NMC, UMT and Vicra both improved image resolution, e.g. at frontal cortical regions (arrows). This area usually suffers more rotation-induced motion since it is far from the head’s center of rotation. In figure 5(a), minor visual differences were observed between Vicra and UMT methods. In figure 5(b), UMT and Vicra yield improved lateral cortical structures (arrow) while NMC was blurred. These examples show that UMT can achieve similar motion tracking performance to Vicra in human studies.

**Figure 6.**
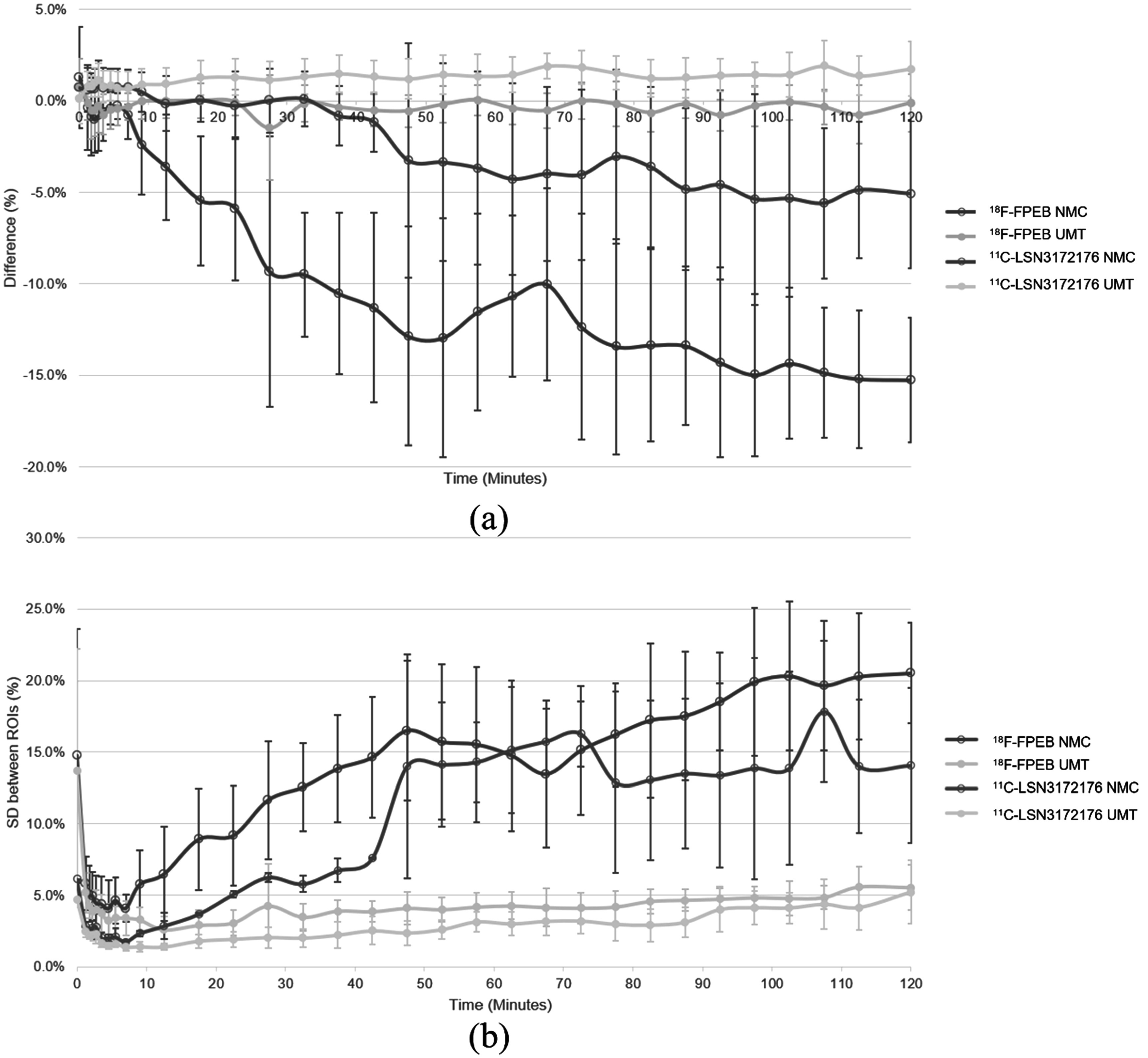
Small ROI mean (a) and SD (b) across ROIs of the percent difference between NMC and UMT versus Vicra over individual scan frames. Error bars illustrate SD across different subjects. NMC: No Motion Correction. SD: Standard deviation. UMT: United Imaging Healthcare Motion tracking system.

Table 2 shows the SUV analysis over 2 h scans for large ROIs for NMC and UMT, with percentage differences reported with respect to Vicra. For ^18^F-FPEB, the average difference between UMT and Vicra was very small (mean ± SD 0.05% ± 0.73%), and the inter-subject SD of UMT-Vicra difference was much less than NMC (5.16% ± 3.11%). For ^11^C-LSN3172176, the average difference between UMT and Vicra was a bit larger (0.84% ± 1.15%), and average SD across subjects between UMT and Vicra was also much less than NMC (3.46% ± 2.82%). For both tracers, grey matter (GM) has higher uptake than white matter, NMC underestimated GM and overestimated white matter while UMT values had a maximum mean difference of 1.18% compared to Vicra. Paired sample t-test showed that NMC absolute bias was significantly greater than UMT (*p* < 0.05) in 12 / 14 ROIs (*p* < 0.1 in the remaining 2 ROIs). Figure 6 shows the SUV analysis for 74 small GM ROIs. In figure 6(a), the mean difference between UMT and Vicra was 0.19% while NMC showed large negative biases. Variability across subjects of the UMT results in both tracers (error bars) was small (mean ± SD 1.11% ± 0.40%). In figure 6(b), for the whole period, the corresponding SD of the % differences across ROIs was also low.

**Table 2.** Brain ROI analysis.

Tracer	^18^F-FPEB		^11^C-LSN3172176	
Brain ROI	NMC bias	UMT bias	NMC bias	UMT bias
Amygdala	−7.87% ± 7.80%	**0.44% ± 1.62%**	−3.26% ± 6.85%	**0.00% ± 2.60%**
Caudate	−23.25% ± 14.67%	**1.36% ± 3.49%**	−8.62% ± 11.66%	**2.96% ± 2.86%**
Cerebellum cortex	−0.90% ± 3.19%	**0.85% ± 0.58%**	**0.98% ± 3.69%**	0.99% ± 0.56%
Frontal	−13.82% ± 5.44%	**−0.64% ± 0.86%**	−4.65% ± 4.49%	**1.46% ± 0.98%**
Hippocampus	−6.93% ± 3.70%	**−0.83% ± 0.79%**	−2.30% ± 6.21%	**0.43% ± 1.99%**
Insula	−10.24% ± 5.05%	**−0.39% ± 1.41%**	**0.40% ± 1.11%**	0.83% ± 0.91%
Occipital	−3.23% ± 9.96%	**0.48% ± 2.06%**	0.73% ± 2.01%	**3.04% ± 2.45%**
Parietal	−9.07% ± 4.41%	**−1.21% ± 0.63%**	11.90% ± 9.68%	**−0.63% ± 1.90%**
Putamen	−6.59% ± 3.71%	**0.02% ± 1.93%**	−1.39% ± 3.97%	**1.22% ± 1.16%**
Temporal	−10.53% ± 14.82%	**0.09% ± 2.27%**	−3.27% ± 2.63%	**1.06% ± 1.37%**
Thalamus	−9.11% ± 5.11%	**−0.27% ± 1.08%**	−3.24% ± 2.48%	**1.65% ± 0.78%**
Cerebellum white matter	1.34% ± 4.58%	**1.07% ± 0.50%**	−1.33% ± 3.69%	**0.12% ± 0.56%**
Cerebral white matter	6.78% ± 4.09%	**0.36% ± 0.83%**	1.64% ± 0.64%	**−0.68% ± 0.34%**
Pallidum*	16.86% ± 4.65%	**−0.70% ± 0.96%**	11.90% ± 9.68%	**−0.63% ± 1.90%**

Grey matter average	−9.23%	**−0.01%**	−1.16%	**1.18%**
Grey matter SD	5.56%	**0.73%**	4.90%	**1.06%**
White matter average	4.06%	**0.72%**	0.16%	**−0.28%**
White matter SD	2.72%	**0.35%**	1.48%	**0.40%**

No motion correction (NMC) and UIH motion tracking (UMT) bias values shown as average ± standard deviation of the % difference with respect to Vicra values across all subjects. Paired sample t-test shows that NMC absolute bias is significantly greater than UMT (*p* < 0.05) in 12/14 ROIs (*p* < 0.1 in the remaining 2 ROIs). SD: standard deviation.

### Motion correction evaluation

**Figure 7.**
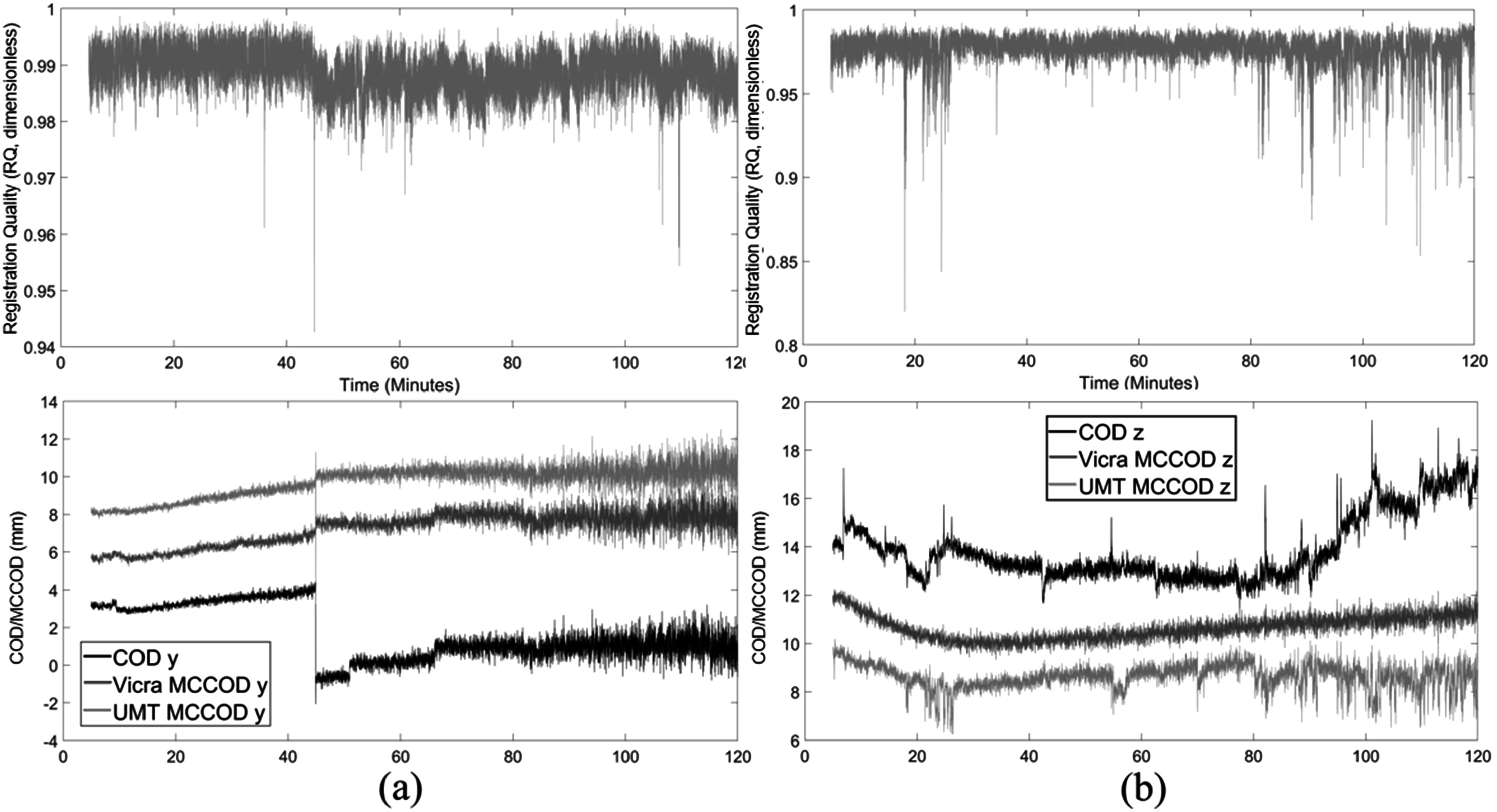
Motion data of one ^11^C-LSN3172176 study (a) and one ^18^F-FPEB study (b). Top: UMT registration quality (*RQ*, dimensionless) data, bottom: COD and MCCOD (mm) with Vicra and the UMT data. Motion data are shown in the *Y* and *Z* directions for (a) and (b), respectively (motion was minimal in other directions). In the bottom panels, the curves were shifted vertically for clarity. Spikes in (b) relate to facial expressions. MCCOD is not shown for the first 5 min because of the rapid change in tracer distribution. COD: Centroid of Distribution. MCCOD: motion-corrected COD. UMT: United Imaging Healthcare Motion tracking system.

Figure 7 shows the UMT *RQ* metric and COD/MCCOD results of one ^11^C-LSN3172176 (figure 7(a)) and one ^18^F-FPEB subject (figure 7(b)); the COD/MCCOD data are shown for the one direction with most prominent motion (there was minimal motion in other directions). In figure 7(a), the proportion of time with *RQ* below 0.97 was 0.03%. The most pronounced spike in the *RQ* curve was at ∼45 min corresponding to a clear motion, as seen in the COD graph in the *Y* direction. Other spikes in *RQ* did not show corresponding movement in the COD graph, perhaps due to non-rigid movements (see Discussion). From 45 to 80 min, there were three head motions from COD-Y, and UMT visually outperformed Vicra in correcting these motions with smoother MCCOD results. Overall, Vicra (blue) and UMT (orange) MCCOD tracers were generally consistent, with increasing fluctuation with time expected due to the ^11^C half-life. In terms of ROI accuracy, the motion correction quality for UMT and Vicra were comparable with average small GM ROI percent difference of 0.18% ± 1.11% in this subject.

Figure 7(b) shows the UMT *RQ* and *Z* direction COD/MCCOD results of one ^18^F-FPEB subject who showed more pronounced movements. From 15–30 and 80–120 min, the *RQ* curve showed numerous spikes (the proportion of time with *RQ* below 0.97 was 10.65%) and COD-Z showed some matching displacements during these periods. Other spikes in *RQ* were caused by facial movement, e.g. mouth breathing (see Discussion) and these were reflected in the UMT motion correction quality from MCCOD. In this case, Vicra outperformed UMT, and the average small GM ROI difference between UMT and Vicra reconstructions was larger (−1.16% ± 3.61%).

## Discussion

In this study, we evaluated the UMT, a commercial markerless head motion tracking system against the Vicra marker-based system. The UMT system yielded similar results as Vicra in both phantom and human point source studies. We then applied UMT motion tracking to 12 human studies and compared reconstruction quality with Vicra-based correction using a common reconstruction platform (Jin *et al* 2013a). For ^18^F-FPEB and ^11^C-LSN3172176, the proposed method achieved comparable reconstructed images and ROI results as compared to Vicra. To evaluate the UMT motion correction quality, we proposed a camera-based metric called registration quality (*RQ*) and verified the feasibility of *RQ* via an existing PET-based motion evaluation method, MCCOD. Note that MCCOD is not applicable in the first minutes postinjection because of the rapid dynamic changes in tracer distribution, whereas *RQ* is able to evaluate the motion tracking performance throughout the scan. Furthermore, *RQ* is capable of assessing performance across all tracers, while MCCOD is dependent on the tracer distribution and the count level. However, RQ relies on the point cloud registration, which limits its applicability to other motion tracking methods.

To use the Vicra system, a tool must be firmly attached to the subject’s head. UMT yielded similar motion tracking results without an attached marker. Generally, markerless motion tracking can enhance the subject’s comfort and simplify operator procedures. In addition, the Vicra tool slippage and wobbling can cause motion tracking errors (Lu *et al* 2020, Sun *et al* 2022a). However, there are still many steps necessary to fully automate and validate the UMT system, including improving the face segmentation and handling non-rigid facial motion. Segmentation of the reference point cloud is needed for accurate motion tracking, and we are currently developing an automatic segmentation algorithm. For facial expression changes, non-rigid motions currently produced tracking errors due to the use of rigid registration. Future work will use camera-based metrics such as *RQ* to detect facial expression changes.

Compared with data-driven methods such as COD and short frame registration (Revilla *et al* 2022, Spangler-Bickell *et al* 2022), camera systems such as UMT should provide better performance in the first minutes postinjection because the rapid change in distribution affects performance of these data-driven methods. In addition, the time resolution of data-driven methods must be optimized, unlike the 30 Hz sampling of UMT.

The goal for the UMT system is its application to the NeuroEXPLORER (NX) brain PET system (Carson *et al* 2021) which should provide useable spatial resolution better than 2 mm with ultra-high sensitivity. In this study, we attached point sources to a phantom and human head and evaluated motion tracking performance based on FWHM analysis. By employing this methodology, we have the ground truth reference from the static time period. In the point source study, the average FWHM difference between reference and UMT is 0.21 ± 0.35 mm, which suggests that UMT may have enough accuracy to track head motion during NX scans without significant loss of resolution. We will include continuous motion in phantom studies in future evaluation on the NX. Furthermore, we evaluated quantitative results on the mCT system and found excellent agreement in ROI results compared to Vicra (table 2). This work will be repeated on the NX and compared with image-based MC such as post reconstruction image registration (Jin *et al* 2013b) because exceptional accuracy in motion correction will be necessary to operate at higher spatial resolution. For the NX, the UMT system will be used without the Vicra system, so an objective evaluation metric for MLMT is important. To assess the quality of the UMT motion data, we proposed the *RQ* metric, which reflects ICP registration accuracy as the fraction of points in the point cloud that are accurately registered to the reference point cloud. Our approach is to apply the *RQ* metric to gate the listmode data, i.e. to remove listmode data when *RQ* detects poor registrations such as the spikes in figure 7. Only count data during periods with high *RQ* will be included in EBE reconstruction and the effective duration of each frame will be adjusted for quantitative reconstruction. In the future, we will evaluate the impact of various *RQ* thresholds (*d*) for gating on the NeuroEXPLORER.

Here, we used the MCCOD method and the proposed *RQ* metric to evaluate the quality of motion correction data. We found that some spikes in the *RQ* trace did not align with displacements of the UMT MCCOD curve. These effects may have been caused by facial expressions. To characterize these effects, we examined the point clouds during periods of low *RQ*. Supplemental figure 3 illustrates the UMT point cloud of a subject with varying facial expressions. Supplemental figure 3(b) corresponds to a spike in the *RQ* curve at 104 min in figure 7(b). The point clouds from these time periods revealed mouth breathing, which led to facial deformation that impacted UMT data. In addition, image-based metrics such as mutual information and cross correlation can also be applied between reference and motion-corrected dynamic frames using different motion correction methods to assess MC quality in the final images, where higher mutual information and cross correlation values correspond to better accuracy (Keller *et al* 2012).

## Conclusion

We evaluated a commercial markerless head motion tracking system against the Vicra system using radioactive point sources on a phantom and a human head as well as on dynamic clinical PET data. UMT outperformed Vicra in the phantom study and achieved comparable results in human point source studies. In twelve human PET studies, UMT achieved comparable results to Vicra, demonstrating promising clinical potential for markerless motion tracking. We proposed a built-in metric called *RQ* for motion tracking evaluation of UMT and compared it with a motion correction evaluation method (MCCOD) which uses PET raw data. Our feasibility data shows that the *RQ* metric is useful in MC evaluation and can detect some facial expression changes. Future work will include evaluation using the NeuroEXPLORER with multiple tracers including ^18^F-FDG.

## Data Availability

The data cannot be made publicly available upon publication because they contain sensitive personal information. The data that support the findings of this study are available upon reasonable request from the authors.

## Disclosure

This work was funded by The National Institutes of Health (NIH) Grants U01EB029811 and R21EB028954. Its contents are solely the responsibility of the authors and do not necessarily represent the official view of NIH. No other potential conflicts of interest relevant to this article exist.
